# GAPDH, β-actin and β2-microglobulin, as three common reference genes, are not reliable for gene expression studies in equine adipose- and marrow-derived mesenchymal stem cells

**DOI:** 10.1186/s40781-015-0050-8

**Published:** 2015-05-07

**Authors:** Fatemeh Nazari, Abbas Parham, Adham Fani Maleki

**Affiliations:** Division of Physiology, Department of Basic Sciences, Veterinary Faculty, Ferdowsi University of Mashhad, Mashhad, Iran; Embryonic and Stem Cell Biology and Biotechnology Research Group, Institute of Biotechnology, Ferdowsi University of Mashhad, Mashhad, Iran

**Keywords:** GAPDH, β-actin, B2M, Reference gene, Mesenchymal stem cells, Equine

## Abstract

**Background:**

Quantitative real time reverse transcription PCR (qRT-PCR) is one of the most important techniques for gene-expression analysis in molecular based studies. Selecting a proper internal control gene for normalizing data is a crucial step in gene expression analysis via this method. The expression levels of reference genes should be remained constant among cells in different tissues. However, it seems that the location of cells in different tissues might influence their expression. The purpose of this study was to determine whether the source of mesenchymal stem cells (MSCs) has any effect on expression level of three common reference genes (GAPDH, β-actin and β2-microglobulin) in equine marrow- and adipose- derived undifferentiated MSCs and consequently their reliability for comparative qRT-PCR.

**Materials and methods:**

Adipose tissue (AT) and bone marrow (BM) samples were harvested from 3 mares. MSCs were isolated and cultured until passage 3 (P3). Total RNA of P3 cells was extracted for cDNA synthesis. The generated cDNAs were analyzed by quantitative real-time PCR. The PCR reactions were ended with a melting curve analysis to verify the specificity of amplicon.

**Results:**

The expression levels of GAPDH were significantly different between AT- and BM- derived MSCs (p < 0.05). Differences in expression level of β-actin (P < 0.001) and B2M (P < 0.006.) between MSCs derived from AT and BM were substantially higher than GAPDH. In addition, the fold change in expression levels of GAPDH, β-actin and B2M in AT-derived MSCs compared to BM-derived MSCs were 2.38, 6.76 and 7.76, respectively.

**Conclusion:**

This study demonstrated that GAPDH and especially β-actin and B2M express in different levels in equine AT- and BM- derived MSCs. Thus they cannot be considered as reliable reference genes for comparative quantitative gene expression analysis in MSCs derived from equine bone marrow and adipose tissue.

## Background

Mesenchymal stem cells (MSCs) are one of the best candidates for cell therapy and regenerative medicine because of their self-renewal, multilineage differentiation, immunomodulatory capabilities and limited tendency to tumorogenesis [1,2]. Many studies have been reported the application of MSCs for treatment of equine musculoskeletal disorders [3,4]. Equine MSCs could be isolated from different sources, including bone marrow, adipose tissue, and umbilical cord [5,6]. Among the sources of equine MSCs that have been isolated so far, bone marrow and adipose tissue are the main sources in clinical trials for treating equine orthopedic disorders [7]. Although MSCs- derived from various sources have similarities, some differences have been reported in biological, proliferative, immunological and differentiative characteristics [8]. MSCs are different in gene expression profile [9,10] and microarray analyses of stemness markers in equine MSCs have shown differences in molecular phenotypes [11]. These differences lead to different functional potentials based on anatomical location [12]. Different characteristics of MSCs can be detectable at transcriptional or translational levels. As a limited specific monolclonal antibodies are available for phenotyping of equine MSCs using immunostaining and flowcytometry techniques, comparative gene expression analysis on mRNA level using quantitative real time- polymerase chain reaction (qRT-PCR) has a great value [13,14].

qRT-PCR is a popular mean to evaluate mRNA expression level and a sensitive and accurate technique for amplification of mRNA [15]. In this method, amplicon accumulation is measured by intercalating dyes such as SYBR Green I [16,17]. However, it is essential to control for errors between samples when measuring RNA expression. Therefore, coincident measurement of a reference gene- also called housekeeping gene- should be used for the normalization of target gene expression data. There are some various reference genes which are involved in different processes in the cells [18].

In the relative quantitative RT-PCR method, an appropriate reference gene should be considered for accurate quantification of RNA expression because the cycle thresholds (C_T_) of the target genes are compared to the reference gene(s) [19]. The expression levels of reference genes should be remained constant between the cells isolated from different tissues [20], otherwise the normalization to varying internal references can result to increased errors [16]. Several genes have been used as housekeeping genes, including β2-microglobulin (B2M), glyceraldehyde 3-phosphate dehydrogenase (GAPDH), β-actin (ACTB), hypoxanthine phosphoribosyl transferase (HPRT) and ribosomal RNA (18 s and 28 s rRNA) [18,21]. From these genes, β-actin, GAPDH and B2M are used most frequent as reference genes in comparative gene analysis in researches on equine marrow- and adipose- derived MSCs [22-24]. GAPDH catalyzes the oxidative phosphorylation of glyceraldehyde 3-phosphate to 1,3-bisphosphoglycerate during glycolysis as well as the reverse reaction in gluconeogenesis. β-actin is one of the cytoskeletal actins that are involved in cell motility, structure and integrity [18,25]. B2M is a structural protein and a component of MHC-I molecule. B2M has positive or negative effect on cell proliferation depending on cell type [26,27]. It seems that cell source and experimental treatment can affect the expression level of housekeeping genes [28]. Therefore, it is important to evaluate the stability of housekeeping genes expression between MSCs- derived from different sources.

The aim of this study was to investigate whether the source of mesenchymal stem cells (MSCs) has any effect on expression level of three common reference genes (GAPDH, β-actin and B2M) in equine marrow- and adipose- derived undifferentiated MSCs and consequently their reliability for comparative qRT-PCR.

## Methods

### Cell isolation, culture and expansion

The experimental protocols were approved by the Committee of Ethics and Animal Welfare of the School of Veterinary Medicine and Animal Science, Ferdowsi university of Mashhad, Iran. Bone marrow (BM) and adipose tissue (AT) samples were collected from three healthy mares aged 3, 6 and 10 years old. BM samples were aspirated using a Jamshidi needle with 1000 IU of sodium heparin/ml. Mononuclear cells were isolated by gradient centrifugation on Histopaque®-1077 (Sigma-Aldrich) for 30 min at 400 g. The cells were rinsed twice with Dulbecco’s phosphate buffered saline (DPBS^−^, Gibco) and plated at 8 × 10^5^ cells/cm2 in 25 cm^2^ flasks in growth medium consisting of high glucose Dulbecco’s Modified Eagle’s Medium (DMEM, Sigma-Aldrich) supplemented with 10% Fetal Bovine Serum (FBS), and 1% Streptomycin/Penicillin and 0.1% amphotericin.

Samples of adipose tissue were collected from gluteal region. The stromal vascular fraction (SVF) was immediately isolated by digestion with 0.1% of collagenase (Type I, Sigma–Aldrich) supplemented with 1% Bovine Serum Albumin (BSA), in an incubator at 37°C for 2 hours. The final solution was centrifuged at 600 g for 5 minutes and the cell-containing pellet was resuspended. The cells were washed twice with DPBS^−^ and seeded at 8 × 10^4^ cells/cm^2^ in 25 cm^2^ flasks in growth medium similar to bone marrow- derived cells.

Cells were incubated at 37°C at 5% CO2 until reaching approximately 80% confluence. The cells were then treated with Tryple enzyme (Invitrogen) and passaged until 3rd passage (P3). P3 cells (3 × 10^6^) were transported to microtubes and stored at −80°C for total RNA extraction.

### Cell characterization

To characterize isolated cells at P3, they were examined for triliniage differentiation capacity (osteogenic, chondrogenic and adipogenic) using defined media. In addition, expression of specific cell surface markers including CD29, CD90, CD34 and MHC-II were investigated.

### RNA extraction, quality analysis and cDNA synthesis

Frozen cells (stored at −80°C) were used for RNA extraction. Total RNA of each sample (containing 2 million cells) was extracted with high pure column RNA extraction kit according to manufacturer’s instructions (Roche, Germany). The RNA concentration and quality was determined by spectrophotometry (NanoDrop Technologies) and gel electrophoresis, respectively. Total extracted RNA was used for cDNA synthesis. Total RNA was treated with RNAse–free DNAse I during extraction phases. 1 μg of RNA was reverse transcribed to cDNA using Accupower kit (South Korea).

### Real-time quantitative PCR

Primer sets of GAPDH and B2M were designed on different exons (intron-spanning) and of β-actin on one exon were designed using Primer Premier V.5 (Premier Biosoft International, Palo Alto, CA, USA), according to the parameters required for the SYBR Green Real-Time PCR [29]. Primer characterization, accession numbers for equine mRNA sequences and length of amplicon are shown in Table 1. The generated cDNAs were analyzed by quantitative real-time PCR. 25 μl reactions were carried out in triplicate in a qRT-PCR cycler (QIAGEN Rotor-Gene 6000 Real-Time Thermal Cycler) using SYBR Green Master Mix (thermo scientific, USA). cDNA samples were diluted 2 times and 2 microlitre of cDNA were used in each reaction along with 200 nM of forward and reverse primers. The cycling conditions were one initial cycle of 94°C for 5 min followed by 40 cycles of 94°C for 30 s, annealing temperature (56.5°C) for 45 s and 72°C for 60 s. The program was ended with a dissociation curve analysis to verify the product and identify the presence of spurious PCR bands or primer dimers. In each experiment, negative control and RT minus sample were used to check template contamination and genomic DNA contamination. Moreover, PCR products were run on 1% agarose gel to confirm specificity of amplification. C_T_ was automatically determined by the Optical System Software (Rotor-Gene Q series software) and these data were exported for further analyses.

**Table 1:** **Information of evaluated internal control genes and their primer sequences**

**Reference gene**	**Accession number**	**function**	**Forward primer**	**Reverse primer**	**Amplicon size (bp)**
Glyceraldehyde-3- phosphate dehydrogenase (GAPDH)	NM_001163856	Oxidoreductase in glycolysis and gluconeogenesis	TGTCATCAACGGAAAGGC	GCATCAGCAGAAGGAGCA	183
β-actin	NM_001081838	Cytoskeletal structural protein	GGGCATCCTGACCCTCAAG	TCCATGTCGTCCCAGTTGGT	63
Β2-microglobulin (B2M)	NM_001082502	A component of MHC class I molecule	CGAAGGTTCAGGTTTACTCACG	ATTTCAATCTCAGGCGGA	97

### Data analysis

C_T_ values of 3 samples in each group (with 3 replicates) were analyzed with *t-test* using SPSS.16 software. To calculate the average fold change in GAPDH, β-actin and B2M expression in AT- MSCs compared to BM- derived MSCS, the mean CT values was calculated as 2^-CT^. Then, average fold change was obtained by division of mean of AT- MSCs value to the mean of BM- derived MSCs value [30].

## Results

### Isolated cells from both sources were characterized as MSCs

Minimal criteria for identification of isolated mesenchymal stem cells were evaluated [14,31,32]. Isolated cells were plastic adherent with a fibroblast-like phenotype. They showed the pattern of mesenchymal markers expression (CD29, CD44, and CD90) and the lack of expression of MHC-II and CD34 (as hematopoietic marker). In addition, cellular differentiation assays demonstrated the chondrogenic, adipogenic and osteogenic potential of the isolated cells.

### RNA integrity and purity

**Figure 1:**
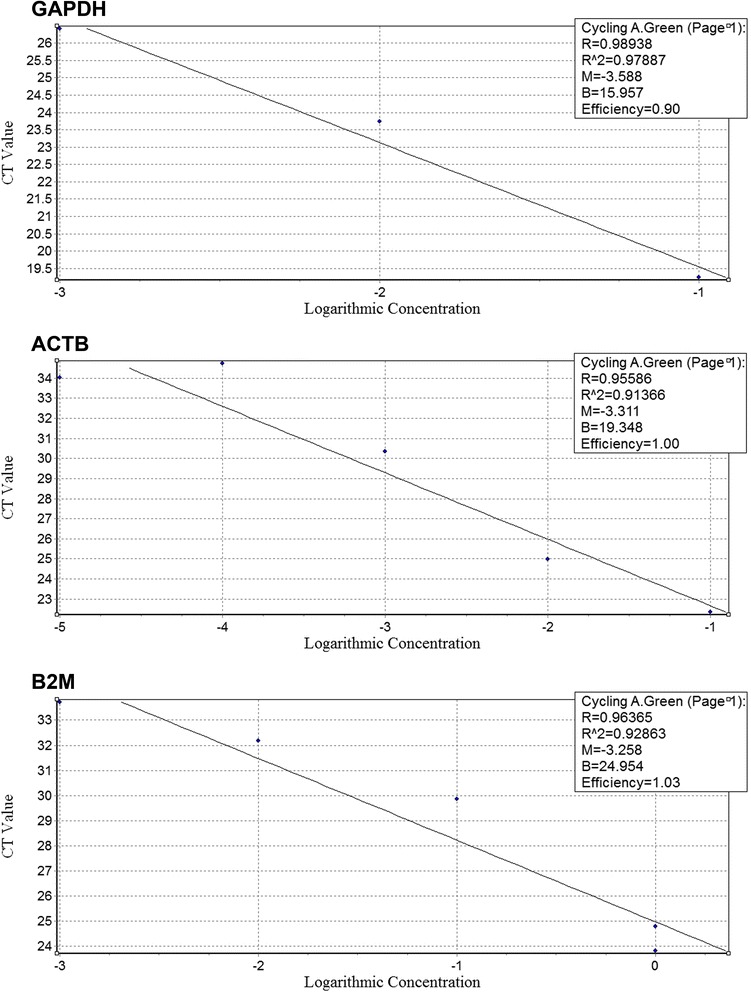
Standard curve for GAPDH, ACTB and B2M genes.

All RNA samples had absorbance values at 260 and 280 nm (A260/280) and 260 and 230 nm (A260/230) 2.0–2.2 and 1.8–2.2, respectively. Gel electrophoresis of RNA samples showed two clear bands related to rRNA (28 s and 18 s) along with 5 s band which confirmed the quality of RNA. There was no evidence of contaminating genomic DNA in all runs. Moreover, GAPDH primers amplified fragments 418 bp and 183 bp from DNA whereas amplified only 183 bp fragment from cDNA. No 418 amplicon was observed in gel electrophoresis of PCR products. Likewise, primers were validated by amplification efficiencies (*E* = 10^-1/slope^) of 100% ± 10% and the efficiency of reactions was 1.90, 2 and 2.03 for GAPDH, β-actin and B2M respectively (Figure 1).

### Expression levels of GAPDH mRNA in equine marrow- and adipose- derived MSCs

**Figure 2:**
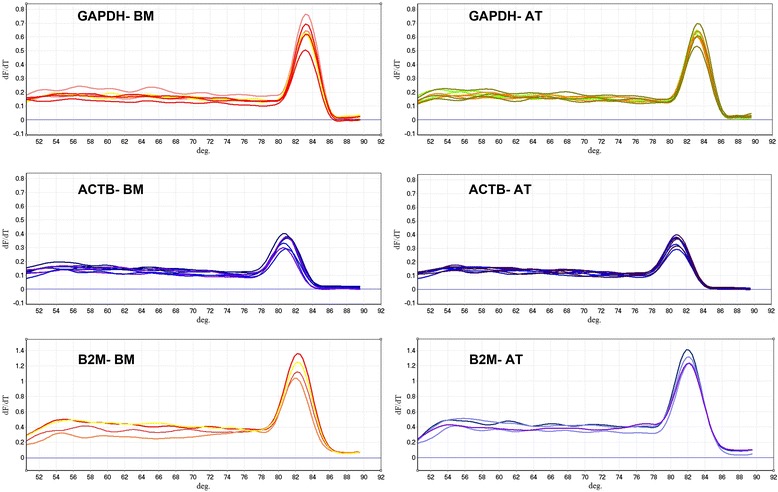
Derivative melting-curve analysis using SYBR Green to identify the dissociation temperature of reference gene amplicons and the specificity of RT-PCR reactions using total RNA from equine marrow- and adipose derived MSCs. The Optical System Software plotted the rate of change of the relative fluorescence units with time (dF)/dT) on the Y-axis versus the temperature (°C) on the X-axis, which peaks at the melting temperature (Tm). Similar peaks indicate no contamination and primer–dimer artifact. BM = Bone marrow; AT = Adipose tissue; MSCs = Mesenchymal stem cells.

**Figure 3:**
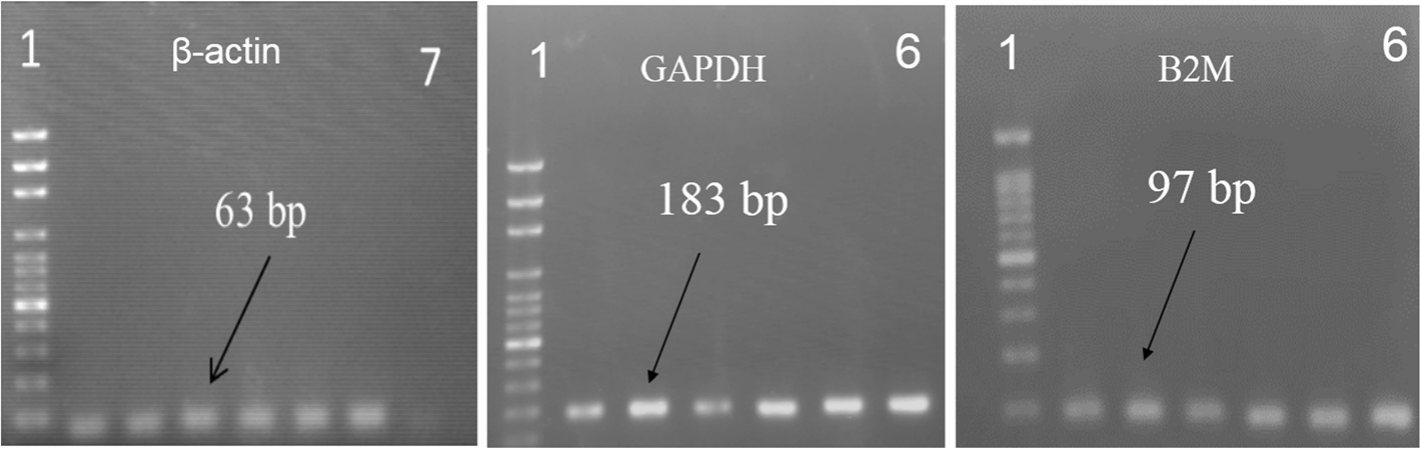
Representative ethidium bromide-stained gel electrophoresis confirmed the size of all the PCR products generated was at 183 bp for GAPDH, 63 bp for β-actin and 97 bp for B2M. First (1) lane is 100-bp DNA marker, 6 next lanes are bone marrow (1–3) and adipose tissue (4–6) samples and the last lane (7) is negative control.

**Figure 4:**
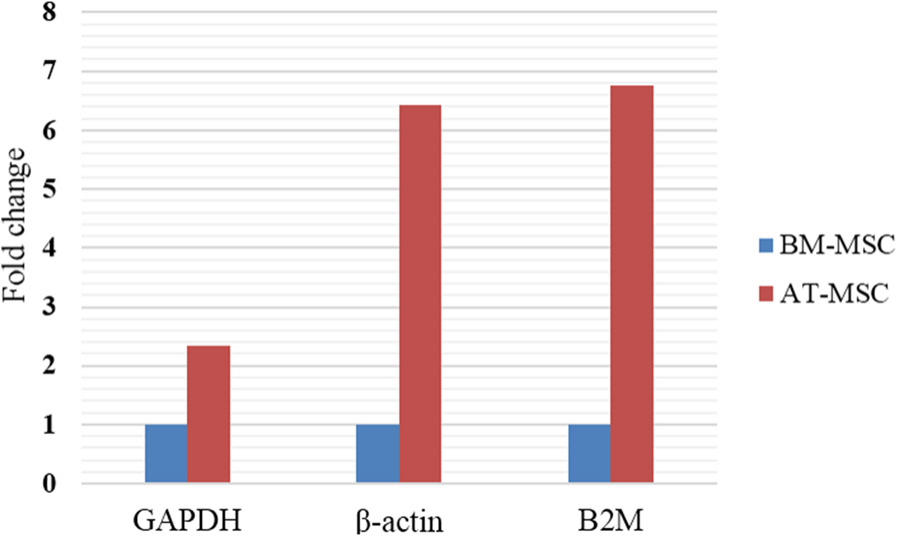
The fold change of GAPDH, ACTB and B2M expression in adipose (AT)- derived MSCs compared with bone marrow (BM)- derived MSCs. It was 2.38, 6.76 and 7.76 for GAPDH, β-actin and B2M in AT- MSCs compared with BM- MSCs, respectively. The mean C_T_ values are calculated as 2^-CT^, then fold change is obtained.

Fluorescence was measured during each PCR cycle and the amount of fluorescence was proportional to the amount of the PCR product. Actually, CT value is inversely proportional to the total RNA concentration isolated from MSCs, hence C_T_ values for BM-MSCs were greater than AT-MSCs ones. The melting curve analysis showed the specificity of the GAPDH primers (Figure 2). Subsequently, the agarose gel electrophoresis was performed to confirm the size and purity of amplification products in various samples (Figure 3). The mean ± SD of C_T_ values for GAPDH expression in AT-MSCs and BM-MSCs is shown in Table 2. Statistical analysis showed that the expression levels of GAPDH were significantly different between AT- and BM- derived MSCs (p < 0.05). The expression level of GAPDH in AT-derived MSCs compared to BM-derived MSCs was 2.38 times (Figure 4).

**Table 2:** **Threshold cycles (C**_**T**_ **) of GAPDH, β-actin and B2M in equine bone marrow- and adipose tissue- derived MSCs**

**Gene**	**Case N.**	**Replicates**	**BM-MSCs**		**AT-MSCs**	
			**C**_**T**_ **mean**	**SD**	**C**_**T**_ **mean**	**SD**
GAPDH	3	3	19.11^a^	2.52	16.19^b^	0.49
ACTB	3	3	21.97^a^	2.21	18.05^b^	0.70
B2M	3	3	22.35^a^	3.14	17.66^b^	1.09

Letters (a, b) represent significant differences in expression levels of GAPDH (P < 0.05), β-actin (P < 0.001) and B2M (P < 0.006) between BM- and AT-derived MSCs. BM = Bone marrow; AT = Adipose tissue; MSCs = Mesenchymal stem cells.

### Expression levels of β-actin mRNA in equine marrow- and adipose- derived MSCs

Similar to GAPDH, fluorescence was measured during each PCR cycle for amplifying ACTB amplicon. Again, each run was completed with a melting curve analysis to confirm the specificity of amplification and lack of primer dimmers (Figure 2). Further analysis by agarose gel electrophoresis confirmed the melt curve analysis (Figure 3). The mean ± SD of C_T_ values for expression of β-actin in AT-MSCs and BM-MSCs is shown in Table 2. Statistical analysis showed that differences in expression levels of β-actin in AT- and BM- derived MSCs were significant (p < 0.001). Actually, the expression level of β-actin in AT-derived MSCs compared to BM-derived MSCs was 6.76 times (Figure 4).

### Expression levels of B2M mRNA in equine marrow- and adipose- derived MSCs

cDNA was amplified by qPCR using B2M primers with a melting curve at the end of amplification (Figure 2). Mean ± SD of quantification Cycle (C_T_) values was determined (Table 2). Statistical analysis indicated significant difference between averages of C_T_ values of two groups (p < 0.006). A single peak in melting curve and lack of nonspecific bands in gel electrophoresis of qPCR products demonstrated that one product was amplified (Figure 3). The expression level of B2M in AT-derived MSCs compared to BM-derived MSCs was 7.76 times (Figure 4).

## Discussion

BM-derived MSCs are widely investigated but because of high proliferation and less invasive access of AT-derived MSCs, this source is more attractive [33,34]. It is suggested that the location of MSCs in various tissues results in some differences in their characteristics [6,9,10]. Comparative gene expression analysis is one of the suitable methods to clarify their differences [22]. Absolute and relative methods are two ways for quantification of gene transcripts. To study changes in gene expression levels in terms of quantity, the relative method is used, which present the data of target gene relative to internal control gene (s) [30,35]. GAPDH, β–actin and B2M are frequently used as endogenous controls for quantitative RT-PCR analysis because their expression seems to be consistent at different time points and various experimental manipulations [20,25,36,37]. In contrast, our finding showed that the expression levels of GAPDH and especially β–actin and B2M were significantly different between AT- and BM- derived MSCs. Moreover, the expression levels of both genes in AT-MSCs were much more than BM-MSCs. So, it seems that the location of MSCs cells could affect the expression levels of these genes.

Recently several scientific reports which are working on different research fields about equine and especially equine MSCs have used common housekeeping genes as reference [5,23,24,38,39]. Many studies have tried to introduce a reasonable method to find a reliable reference gene. The stability of reference gene expression is the definition of endogenous control and it is essential to finding the stable one(s) to every experimental condition or tissue location in the body [24,40,41]. Some studies in the field of equine MSCs have used GAPDH, β-actin and B2M as internal control genes without presenting any data regarding the stability of these genes [5,22,23], whereas some studies have confirmed the instability of their expression which is in agreement with our findings [42,43].

Transcription rate of GAPDH, β-actin and B2M can be influenced by a number of factors such as cellular proliferation. The β-actin functions in cellular shape and anchorage [44]. GAPDH, in addition to its role in glycolytic processes, has been involved in membrane transport, microtubule assembly, protein phosphotransferase/kinase reactions, the translational control of gene expression and DNA replication [45]. B2M stimulate cell proliferation in stromal cells so that its over expression and knockdown affect proliferation of MSCs [46]. So, cellular proliferation can induce upregulation of GAPDH, β-actin and B2M gene expression [47]. In agreement with Burk et al. [10], we previously found that the proliferative capacity of AT-MSCs is more than BM-MSCs [31,32] which can explain the greater expression levels of GAPDH, β-actin and B2M in AT-MSCs compared to BM-MSCs.

## Conclusion

Although many studies in the field of equine MSCs commonly have used GAPDH, β–actin and B2M as simple internal genes, it seems that their expression levels in MSCs isolated from different tissues is not stable and they could not be considered as suitable reference genes for comparative gene analysis studies. Thus, it is necessary to find and introduce proper reference genes for comparative gene expression analysis studies in equine MSCs isolated from bone marrow and adipose tissue.

## References

[1] Alves AG, Stewart AA, Dudhia J, Kasashima Y, Goodship AE, Smith RK (2011). Cell-based therapies for tendon and ligament injuries. Vet Clin North Am Equine Pract.

[2] Li Y, Yu X, Lin S, Li X, Zhang S, Song YH (2007). Insulin-like growth factor 1 enhances the migratory capacity of mesenchymal stem cells. Biochem Biophys Res Commun.

[3] Smith RKW, Korda M, Blunn GW, Goodship AE (2003). Isolation and implantation of autologous equine mesenchymal stem cells from bone marrow into the superficial digital flexor tendon as a potential novel treatment. Equine Vet J.

[4] Nixon AJ, Dahlgren LA, Haupt JL, Yeager AE, Ward DL (2008). Effect of adipose-derived nucleated cell fractions on tendon repair in horses with collagenase-induced tendinitis. Am J Vet Res.

[5] Radtke CL, Nino-Fong R, Gonzalez BPE, Stryhn H, McDuffee LA (2013). Characterization and osteogenic potential of equine muscle tissue–and periosteal tissue–derived mesenchymal stem cells in comparison with bone marrow–and adipose tissue–derived mesenchymal stem cells. Am J Vet Res.

[6] Kern S, Eichler H, Stoeve J, Klüter H, Biebak K (2006). Comparative analysis of mesenchymal stem cells from bone marrow, umbilical cord blood, or adipose tissue. Stem Cells.

[7] Koch TG, Berg LC, Betts DH (2008). Concepts for the clinical use of stem cells in equine medicine. Can Vet J.

[8] Strioga M, Viswanathan S, Darinskas A, Slaby O, Michalek J (2012). Same or not the same? Comparison of adipose tissue-derived versus bone marrow-derived mesenchymal stem and stromal cells. Stem Cells Dev.

[9] Lee R, Kim B, Cho iI, Kim H, Cho iH, Suh K (2004). Characterization and expression analysis of mesenchymal stem cells from human bone marrow and adipose tissue. Cell Physiol Biochem.

[10] Burk J, Ribitsch I, Gittel C, Juelke H, Kasper C, Staszyk C (2013). Growth and differentiation characteristics of equine mesenchymal stromal cells derived from different sources. Vet J.

[11] Al-Nbaheen M, Ali D, Bouslimi A, Al-Jassir F, Megges M, Prigione A (2013). Human stromal (mesenchymal) stem cells from bone marrow, adipose tissue and skin exhibit differences in molecular phenotype and differentiation potential. Stem Cell Rev.

[12] Campioni D, Lanza F, Moretti S, Ferrari L, Cuneo A (2008). Loss of Thy-1 (CD90) antigen expression on mesenchymal stromal cells from hematologic malignancies is induced by in vitro angiogenic stimuli and is associated with peculiar functional and phenotypic characteristics. Cytotherapy.

[13] Radcliffe CH, Flaminio MJBF, Fortier LA (2010). Temporal analysis of equine bone marrow aspirate during establishment of putative mesenchymal progenitor cell populations. Stem Cells Dev.

[14] De Schauwer C, Meyer E, Van de Walle GR, Van Soom A (2011). Markers of stemness in equine mesenchymal stem cells: a plea for uniformity. Theriogenology.

[15] Bustin SA (2000). Absolute quantification of mRNA using real-time reverse transcription polymerase chain reaction assays. J Mol Endocrinol.

[16] Bustin SA, Kubista M, Benes V, Mueller R, Garson JA, Nolan T (2009). The MIQE guidelines:minimum information for publication of quantitative real-time PCR experiments. Clin Chem.

[17] Huggett J, Dheda K, Bustin S, Zumla A (2005). Real-time RT-PCR normalisation; strategies and considerations. Genes Immun.

[18] Vandesompele J, De Preter K, Pattyn F, Poppe B, Van Roy N, De Paepe A (2002). Accurate normalization of real-time quantitative RT-PCR data by geometric averaging of multiple internal control genes. Genome Biol.

[19] Pfaffl MW (2001). A new mathematical model for relative quantification in real-time RT-PCR. Nucleic Acids Res.

[20] Thellin O, Zorzi W, Lakaye B, De Borman B, Coumans B, Hennen G (1999). Housekeeping genes as internal standards: use and limits. J Biotechnol.

[21] Lee PD, Sladek R, Greenwood CMT, Hudson TJ (2002). Control genes and variability: absence of ubiquitous reference transcripts in diverse mammalian expression studies. Genome Res.

[22] Ranera B, Lyahyai J, Romero A, Vazquez FJ, Remacha AR, Bernal ML (2011). Immunophenotype and gene expression profiles of cell surface markers of mesenchymal stem cells derived from equine bone marrow and adipose tissue. Vet Immunol Immunopathol.

[23] Ranera B, Ordovas L, Lyahiah J, Bernal ML, Fernandes F, Remacha AR (2012). Comparative study of equine bone marrow and adipose tissue-derived mesenchymal stromal cells. Equine Vet J.

[24] Zhang YW, Davis EG, Bai J (2009). Determination of internal control for gene expression studies in equine tissues and cell culture using quantitative RT-PCR. Vet Immunol Immunopathol.

[25] Barber RD, Harmer DW, Coleman RA, Clark BJ (2005). GAPDH as a housekeeping gene: analysis of GAPDH mRNA expression in a panel of 72 human tissues. Physiol Genomics.

[26] Josson S, Nomura T, Lin J-T, Huang W-C, Wu D, Zhau HE (2011). b2-microglobulin induces epithelial to mesenchymal transition and confers cancer lethality and bone metastasis in human cancer cells. Cancer Res.

[27] Nomura T, Zhau W-CHE, Josson S, Mimata H, Chung LWK (2014). B2-Microglobulin-mediated Signaling as a Target for Cancer Therapy. Anticancer Agents Med Chem.

[28] Schmittgen TD, Zakrajsek BA (2000). Effect of experimental treatment on housekeeping gene expression: validation by real-time, quantitative RT-PCR. J Biochem Biophys Methods.

[29] Dorak MT (2006). Real-Time PCR.

[30] Schmittgen TD, Livak KJ (2008). Analyzing real-time PCR data by the comparative CT method. Nat Protoc.

[31] Alipour F, Parham A, Kazemi Mehrjerdi H, Dehghani H (2015). Equine adipose-derived mesenchymal stem cells: phenotype and growth characteristics, gene expression profile and differentiation potentials. Cell J.

[32] Zahedi M, Abavisani A, Dehghani H, Kazemi Mehrjerdi H (2013). Isolation and characterization of horse bone marrow mesenchymal stem cells for treatment of joint injuries: an animal model for human studies. Artif Organs.

[33] Russell KC, Lacey MR, Gilliam JK, Tucker HA, Phinney DG, O’Connor KC (2011). Clonal analysis of the proliferation potential of human bone marrow mesenchymal stem cells as a function of potency. Biotechnol Bioeng.

[34] Ragni E, Viganò M, Rebulla P, Giordano R, Lazzari L (2013). What is beyond a qRT‐PCR study on mesenchymal stem cell differentiation properties: how to choose the most reliable housekeeping genes. J Cell Mol Med.

[35] Nolan T, Hands RE, Bustin SA (2006). Quantification of mRNA using real-time RT-PCR. Nat Protoc.

[36] Lupberger J, Kreuzer K-A, Baskaynak G, Peters U, Le Coutre P, Schmidt C (2002). Quantitative analysis of beta-actin, beta-2-microglobulin and porphobilinogen deaminase mRNA and their comparison as control transcripts for RT-PCR. Mol Cell Probes.

[37] Amable PR, Teixeira MVT, Carias RBV, Granjeiro JM, Borojevic R (2013). Identification of appropriate reference genes for human mesenchymal cells during expansion and differentiation. PLoS One.

[38] Hjertner B, Olofsson KM, Lindberg R, Fuxler L, Fossum C (2013). Expression of reference genes and T helper 17 associated cytokine genes in the equine intestinal tract. Vet J.

[39] Sánchez-Matamoros A, Kukielka D, De las Heras AI, Sánchez-Vizcaíno JM (2013). Development and evaluation of a SYBR Green real-time RT-PCR assay for evaluation of cytokine gene expression in horse. Cytokine.

[40] Bustin S, Penning LC (2012). Improving the analysis of quantitative PCR data in veterinary research. Vet J.

[41] Chooi WH, Zhou R, Yeo SS, Zhang F, Wang D-A (2013). Determination and validation of reference gene stability for qPCR analysis in polysaccharide hydrogel-based 3D chondrocytes and mesenchymal stem cell cultural models. Mol Biotechnol.

[42] Glare EM, Divjak M, Bailey MJ, Walters EH (2002). β-Actin and GAPDH housekeeping gene expression in asthmatic airways is variable and not suitable for normalising mRNA levels. Thorax.

[43] Lin J, Redies C (2012). Histological evidence: housekeeping genes beta-actin and GAPDH are of limited value for normalization of gene expression. Dev Genes Evol.

[44] Bursten S, Stevenson F, Torrano F, Lovett D (1991). Mesangial cell activation by bacterial endotoxin. Induction of rapid cytoskeletal reorganization and gene expression. Am J Pathol.

[45] Sirover MA (1999). New insights into an old protein: the functional diversity of mammalian glyceraldehyde-3-phosphate dehydrogenase. Biochim Biophys Acta.

[46] Zhu Y, Su Y, Cheng T, Chung LW, Shi C (2009). β2-Microglobulin as a potential factor for the expansion of mesenchymal stem cells. Biotechnol Lett.

[47] Kim JW, Kim SJ, Han SM, Paik SY, Hur SY, Kim YW (1998). Increased glyceraldehyde-3-phosphate dehydrogenase gene expression in human cervical cancers. Gynecol Oncol.

